# “A degraded man, […] a half-civilized savage”: Shame and Gender in Nineteenth-Century Irish Temperance Literature (1840–1878)

**DOI:** 10.1086/741293

**Published:** 2026-09-01

**Authors:** Fionnula Simpson

## Abstract

In Ireland, nineteenth-century iterations of the temperance movement operated within a politically charged discourse that insisted upon a direct link between Irish identity and ritualized alcohol consumption. This article uses temperance literature, a critically neglected genre of Irish fiction, to demonstrate the adaptation of the “drunken Irishman” stereotype to emerging nineteenth-century medical frameworks using highly gendered imagery and tropes. Drawing on fictional as well as medical works, it demonstrates the ways in which writers like Anna Maria Hall, William Carleton, and Marion Clarke reinforced the idea of the drinker’s shame by positioning the male drinker as embarrassingly emasculated and the female drinker as grotesquely defeminized. It argues that these portrayals of male violence, poverty, and neglectful mothering mask darker anxieties over the threat that the lower classes supposedly posed to the social order, providing an insight into Irish writers’ engagement with ideas of a national predilection for alcohol that supposedly enhanced hypermasculine and effeminate traits.

In 1866, *The Irish Temperance Star* published a short article under the title “Manliness.”^1^ In the sentimental, moralizing tone typical of the temperance genre, its anonymous author mourned the perceived erosion of Irish masculinity due to the purportedly widespread practice of heavy drinking: “What a degradation to hear the voice, which might have been persuasive in truest eloquence, articulating drunken imbecilities, in maudlin, almost unintelligible utterances!”^2^ The author went on to plead with young men to adopt total abstinence, lest their manliness be degraded and they become “brutalized by intemperance.”^3^ The idea that intoxication posed a threat to one’s masculinity was widely propagated in nineteenth-century temperance writing and medical works. Prominent British and Irish doctors often referred to the drinker in terms of emasculation—Charles Wilson, for instance, described patients who suffered from *delirium tremens* as being “thoroughly unmanned,”^4^ Charles Follen Palmer presented almost as self-evident the idea that “inebriety” eroded “moral manhood,”^5^ and Henry Osborne wrote for the *Journal of the Statistical and Social Inquiry Society of Ireland* that drinking “degrades the most generous hearts that beat among men.”^6^ The figure of the emasculated male drinker had a wide-ranging influence over medical and cultural attitudes to drink, and Joanne Begiato and Gale Temple have shown how alcohol was interpreted as a potentially feminizing force in Britain and America, respectively.^7^ However, this article argues that implications of gendered shaming, which as we shall see was an accepted strategy with which to attempt “reform” of an individual’s drinking habits, deserve further attention within the Irish context given the then-prevalent idea that “there was something in the people’s constitution congenial to the excitement of ardent spirits.”^8^

The perceived association between heavy drinking and Irishness was pervasive and long-standing, harking back to the appearance of the bumbling figure of the “Stage Irishman” in Elizabethan drama.^9^ Lower-class Catholic tenant farmers were renowned for their supposedly liberal indulgence in “potheen,” or illegally distilled whiskey, and the landowning Protestant Ascendancy gained a reputation for de-bauched profligacy via their assertion of a raucous masculinity expressed through toasts and tipples.^10^ However, after the 1798 rebellion, in which heavy alcohol consumption was said to have played a crucial role, intoxication came to be regarded as a signifier of specifically lower-class dysregulation and disruption.^11^ The shift in attitudes toward Irish drinking culture can be at least partly explained by the spread of middle-class British values to Ireland. As Norman Tosh and John Vance have both demonstrated, British class-based codes of “gentlemanly” behavior that allowed for and even encouraged displays of heavy drinking among men were gradually eclipsed by a new definition of “manliness,” which meant that self-restraint, independence, and hard work were now regarded as ideal attributes for any man, regardless of his class.^12^ Manliness provided a useful blueprint for male behavior not only because it encouraged industry in a rapidly industrializing society^13^ but also because it encouraged men to exercise self-control over their desires for things like sex, tobacco, and, of course, alcohol.^14^ Thus it was that the figure of the heavy-drinking, hard-shooting, ruddy-faced gentleman who symbolized the eighteenth-century English elite fell from grace and came to be associated with waste and excess.^15^ After the Act of Union in 1801, which amalgamated Britain and Ireland into one political entity, these British norms of respectability were widely adopted by upper-class Protestants in Ireland.

The union between Britain and Ireland was often framed by political commentators in gendered and conjugal terms, with the states occasionally referred to as sister kingdoms but more often as husband and wife—a strategic allegory that served various purposes over the course of several centuries. The Tudors represented Ireland as being in need of good “husbandry” in order to justify their invasion, and, later, British cartoonists depicted a feminized version of Ireland that required protection from the supposedly dangerous Fenians who were in favor of Home Rule.^16^ As Mary J. Corbett has shown, the conjugal trope not only attempted to naturalize national hierarchy but also calls into question essentialist constructions of racial and gender difference.^17^ Following the work of Edward Said, various historians have demonstrated that male subjects of a colony are often racialized, feminized, or cast as “Other” by colonizers who wish to justify their domination, and Joseph Valente convincingly argues that this process also took place in Ireland,^18^ notwith-standing the ambiguity surrounding Ireland’s status as a colony.^19^ According to Valente, nineteenth-century British writers and politicians used the construction of Ireland as “wife” in order to reinforce the notion that the Irish were too irrational, too passionate, and too prone to excess—qualities then regarded as essentially feminine—to govern themselves effectively.^20^ At the same time, the contrasting tropes of the feminized Irishman (who was characterized as emotionally volatile and passionate) and the hypermasculine Irishman (who was supposed to be particularly prone to violence) combined to delegitimize agrarian and Irish Revolutionary Brotherhood agitations.^21^ In temperance writing, drink was portrayed as a substance that enhanced these apparently feminine and hypermasculine traits simultaneously, rendering the drinker “maudlin” and “brutalized” by turns, as the opening extract illustrates.^22^ In fact, the supposed femininity of the Irish Celt was, as David Lloyd has shown, inextricably linked with his characterization as a problematic drinker who transgressed masculine ideals through excess.^23^ Concerns about violent, unruly, intoxicated bodies were particularly linked to fears of lower-class insurrection, and by casting all forms of excess as shameful, temperance writers wielded idealized masculinity as a tool with which to maintain public order.

The racialized, gendered, and politically charged nature of the discourse surrounding the drinking habits of Irish people is particularly significant in light of medical developments related to alcohol that occurred internationally throughout the nineteenth century. While doctors raised concerns about heavy drinking prior to this period—for instance, during the British “Gin Craze” of the 1730s and 1740s^24^—the health impacts of alcohol consumption were increasingly discussed in medical circles as the nineteenth century progressed, and various theorizations of “problematic” drinking emerged that framed the issue in terms of a diseased mind.^25^ As James Nicholls outlines, from the eighteenth century onward, medical writing attempted to redefine “drunkenness” in terms of disease—initially metaphorically, then literally—but they nevertheless frequently drew upon arguments that framed drunkenness as a vice.^26^ Similarly, Virginia Berridge argues that the British medical profession was too closely entwined with the temperance movement in the nineteenth century to make meaningful progress in developing a disease theory that could be distinguished from moralized interpretations of the drinker’s behavior.^27^ While in some ways a concept of alcohol “addiction” was medicalized in Britain during this period, Berridge demonstrates that cure was still regarded as a matter of the individual’s moral reformation rather than medical intervention.^28^ A survey of the medical literature certainly reveals an overwhelming emphasis on the perceived moral failings of the drinker and a prevailing idea that it was possible to shame him into sobriety. Wilson advocated the use of “ironical and contemptuous treatment [… to bring] the drunkard to a sense of the shame of his condition,”^29^ and even Norman Kerr, a Scottish doctor and temperance reformer who elsewhere criticized the medical tendency to stigmatize the drinker as “a good-for-nothing scapegrace,”^30^ referred to “the selfishness, untruthfulness, cunning, and deceit of the alcohol inebriate.”^31^ In Ireland, doctors were influenced by these international trends but also made their own contributions to the study of alcohol, as Alice Mauger has shown.^32^ Displaying a similar attitude to that which can be seen in British medical works, they tended to depict the drinker as an immoral individual who lacked self-restraint,^33^ and in their efforts to rescue the drinker from a habit they regarded as detrimental to both body and soul, they too regarded shame as a powerful motivator. For instance, William Saunders Hallaran, who was superintendent of the Cork Lunatic Asylum ca.1785 to 1825, believed that doctors should attempt to thoroughly “convict [drinkers] of their unworthiness” in order to persuade them into sobriety, although he admitted that they were generally “incorrigible” and tended ultimately to give way to their “insatiable cravings.”^34^

This tendency to hold the drinker up for moral scrutiny as well as medical study can partly be explained by the close relationship between the medical field and the temperance movement. Though the tenets of Irish temperance were rooted in Protestant antidrink campaigns, the most famous iteration of the movement occurred under the leadership of Father Mathew, a charismatic Capuchin priest who advocated total abstinence from alcohol.^30^ By positioning the welfare of the family at the heart of the temperance mission, Father Matthew ensured sobriety was tied to participation in gender roles that became increasingly narrowly defined as the century progressed.^35^ Father Mathew claimed he had recruited five million souls to the cause by 1842—a figure that encompassed over half of the Irish population at the time.^36^ However, as Mauger points out, many individuals who were treated for alcohol-related mental disorders in lunatic asylums had taken the pledge before beginning to drink again.^37^ While it is therefore important not to over-state the actual rate of abstinence in Ireland, it is clear that Father Matthew’s campaign affected a significant shift in attitudes toward drink, with many coming to regard it as a source of shame on both an individual and national level.^38^ The beginning of the Great Famine in 1845 put an end to Father Mathew’s campaign, and the temperance movement did not gain significant traction among Catholics again until 1889, when Reverend James A. Cullen founded the Total Abstinence League of the Sacred Heart.^39^ Unlike Father Mathew’s self-proclaimed political neutrality, Reverend Cullen was a nationalist who regarded temperance as a crucial step toward Irish political freedom.^40^

Temperance institutes became fertile recruiting ground for nationalists, and the widespread adoption of temperance among the lower classes served as a further justification for gaining Irish sovereignty.^41^ Nationalist politicians railed against offensive representations of the stereotypical “drunken Irishman” that were used to justify British domination, and they drew on centuries-old arguments made by antidrink campaigners to perpetuate the notion of liberty attained through sobriety.^42^ Lloyd argues that the nationalist adoption of temperance was part of a wider project that aimed to resist the stereotype of the effeminate Celt by “re-masculinizing the Irish public sphere.”^43^ By the end of the nineteenth century, a distinctly nationalist definition of manliness emerged as a combination of Victorian gender ideals and Roman Catholic praxis,^44^ and men who wished to be perceived as manly were encouraged to practice self-restraint around alcohol. As Diarmaid Ferriter outlines in *A Nation of Extremes* (1998), temperance accordingly became a foundational component of the twentieth-century Irish nationalist movement.^45^ Temperance was therefore used by two different groups to forward two very different political aims, but Protestant reformers and Irish nationalists both accepted the British paradigm of model citizenship that promoted individual self-improvement, temperance, and an emphasis on the nuclear family.^46^

The perceived necessity of establishing the “Irishness” of work by Irish authors has led to what James H. Murphy terms a “secret anxiety” among literary historians that fiction that appears almost indistinguishable in tone or emphasis from British works is “not truly Irish at all, but merely the product of an assimilation to British culture.”^47^ Perhaps it is partly for this reason that Irish temperance literature, with its endorsement of a notably Protestant middle-class morality, has hitherto received scant critical attention, but I argue that the genre provides a useful case study through which to examine the adaptation of the “drunken Irish” stereotype to new medical frameworks via the idealization of middle-class gender norms. In taking this line of argument, I do not attempt to demonstrate a hierarchical influence of medicine over literature or vice versa but rather to highlight the mutually constructive nature of these fields as scholars like Sally Shuttleworth and Lucy Cogan have previously demonstrated.^48^ Accordingly, this article focuses on the gendered and medicalized aspects of temperance literature rather than on the religious dimensions of the genre, following Claire Connolly’s *Cultural History of the Irish Novel* (2011), which seeks to redress what she regards as the literary historical tendency to focus exclusively on the regional, national, and religious features of Irish writing.^49^ While temperance writers were mainly middle or upper class, their work did not necessarily constitute a literal attempt to inculcate Catholic readers with Protestant doctrine, as we can see from William Carleton’s Catholic origins and Marion Clarke’s Presbyterianism.

Although it is often reported that nineteenth-century Irish literature lacks a coherent rhetoric of “progress,” Helen M. O’Connell demonstrates that improvement fiction set out to challenge contemporary conceptions of Ireland as backward and drunken in order to facilitate assimilation with Britain.^50^ Similarly, Niall ó Ciosáin shows how middle- and upper-class Irish authors distributed persuasive literary tracts that displayed the advantages of industry, cleanliness, and sobriety in an attempt to exert control over lower-class populations.^51^ Paternalistic ideas of improvement also provided motivation for the flurry of culturally nationalist periodicals that emerged during a period of relative political stability after Catholic Emancipation (1829) in the 1830s.^52^ The *Dublin Penny Journal*, for example, founded by Caesar Otway and George Petrie in 1832, aimed to provide useful information that would encourage and educate the lower-class Irish reader, and included temperance tales within its pages.^53^ However, it remains unclear whether lower-class individuals whose drinking was regarded as problematic actually read the short stories and novels produced by advocates of the temperance movement. ó Ciosáin argues that improvement literature gained little traction among the Irish peasantry and demonstrates that didactic tracts were mainly sold to middle-and upper-class individuals. He therefore surmises that these tales of lower-class conversion and reform were calculated “to reassure elite purchasers and to convince them of the feasibility of reform of popular culture by persuasion.”^54^ While O’Connell provides evidence that certain improvement tracts were freely distributed among the peasantry,^55^ the absence of definitive evidence for a widespread lower-class readership (and the fact that authors of improvement literature were generally middle or upper class themselves) means that the temperance genre is, as Karen Sanchez-Eppler points out, “better understood as a story domesticity tells about itself to itself.”^56^

These domestic tales not only provide a useful insight into middle- and upper-class attitudes toward the “improvement” of the poor in nineteenth-century Ireland—they also speak to Irish writers’ broader engagement with ideas of a national predilection for alcohol that supposedly enhanced hypermasculine and effeminate traits. In doing so, these authors gave voice to their lurking anxieties about lower class people who drank: that the men were vicious and unproductive and that the women were neglectful mothers. As Stephen Earnshaw notes, “drunkards” in nineteenth-century literature are generally “treated en masse as types rather than unique, individuated souls,”^57^ and Irish temperance writers repeatedly draw on the tropes of the violent male drunkard, the grotesque drunk mother, and their long-suffering, impoverished families that will prove immediately recognizable to those who are familiar with European or American temperance literature. These attempts to impose the prevailing didactic formula on the Irish context are rather unconvincing, as we shall see, but they help to demonstrate how medicine and temperance literature engaged in the co-construction of the supposedly problematic Irish drinker in a way that upheld social and gendered hierarchy. Throughout this article, I explore representations of the drinker in the work of Irish temperance writers who range from the canonical (William Carleton) to the once popular but now obscure (Anna Maria Hall and Marion Clarke). Taking a roughly chronological, broad survey approach to temperance stories written from the 1840s to the 1870s, I outline rhetorical trends that closely align with medicalized ideas surrounding alcohol consumption, demonstrating a prevailing tendency to direct shame toward the drinker in terms of his or her failure to adhere to gender ideals over a relatively broad period.

## The Cost of Drunkenness

The image of the lower-class family rendered destitute by the antics of a heavy-drinking, male head of household emerges as one of the most prolific tropes of the temperance genre. It is possible to argue, as Annemarie McAllister does, that temperance movements granted a degree of economic liberation to lower-class individuals by denormalizing drinking culture and encouraging workers to save their money for something other than the pub.^58^ However, in Ireland, the Great Famine (1845–1852) underscored the falsity of the argument that industry and sobriety could provide the lower classes with a sure safeguard against starvation. This national tragedy, during which approximately one million people died, can be attributed in large part to the prevailing *laissez faire* economic system that provided the British government with justification for their policy of nonintervention.^59^ Despite this grim reality, a survey of the literature reveals that Irish temperance writers portrayed the lower classes as being almost entirely responsible for the issues, like poverty, violence, and child neglect, that were thought to proliferate in their communities, utilizing this shaming rhetoric at a time when the financial cost of dealing with the poor was becoming increasingly burdensome. The introduction of the poor law system in 1838 effected a dramatic increase in the number of asylums and workhouses, but after the Famine, this network proved itself entirely unable to cope with the vast numbers who required assistance.^60^ Alcohol appears to have provided a convenient scapegoat for middle and upper-class authors, who wrote with horrified fascination of the widespread, abject poverty that was, according to the rhetoric of their tales, almost entirely brought about by an unquenchable thirst for intoxicating liquor among lower-class men. Writing for the *Irish Temperance League Journal* in 1865, James Haughton addressed farmers with the bold statement: “But for strong drink, there need not be a pauper in Ireland,”^61^ and, continuing in the same vein, a periodical article published in 1870 lamented the fact that “the intemperate man may now, either at home or in licensed hours in a public house, drink with impunity, to a degree that will involve in ruin not only the moral but the *material* interests of himself and his family” (emphasis added).^62^ It seems that impoverished families, rather than endangered souls, were what truly concerned many temperance reformers over the course of the mid-nineteenth century. Despite their strongly religious overtones, these stories convey the surprising sense that the real horror of drunkenness lies not in its spiritual consequences (although these are inevitably dire) but in its shameful proximity to poverty, destitution, and disease. By arguing that extreme poverty was the inevitable result of excessive indulgence in drink, these writers played on conceptions of the husband as a strong, reliable provider to evoke shame in men who fell short of this masculine ideal, urging them to work not only for their families but also for the economic improvement of Ireland.^63^

The work of Anna Maria Hall (1800–1881), who wrote prolifically about the Irish peasantry throughout her lifetime, can be used to illustrate how the figure of the lower-class male drinker came to personify these concerns. Hall published her tales in several leading Irish magazines, including the *Dublin Literary Gazette*, the *Irish Penny Magazine*, and the *Dublin Penny Journal*, but attracted criticism from Irish reviewers that she pandered to a middle-class, English audience.^64^ It is not difficult to find justification for these comments in Hall’s work, which is patronizingly moralistic and perpetuates offensive stereotypes about Irish people—that they are, for example, content to say “‘sure it was always so’ and ‘it’ll do,’ […] and withstand any changes that indicate and lead to improvement.”^65^ While she laments the fact that “an Irishman is held up as a degraded man, as a half-civilized savage,” her tales do very little to challenge this stereotype.^66^ Hall’s lower-class, drunken men *are* degraded, and they are very often savage, but by the end of the story, their living conditions have been transformed and enriched through their adoption of temperance. Her tales therefore exist both as a reinforcement of and an intended remedy for Ireland’s drunken reputation by demonstrating that Irish men could be redeemed from their supposedly degraded state if they renounced “the old habit” that had made “the name of an Irishman a byword and reproach, instead of a glory and an honour.”^67^

Hall’s tales predictably align with the moral arc commonly presented in temperance literature that emphasizes the idea that the drunkard had only to renounce whiskey and resume work in order to provide abundantly for his family, notwithstanding the socioeconomic constraints that were harsh realities for many lower-class Irish families of the time. In “The Real Temperance Cordial,” for instance, which was published in *The Irish Penny Journal* in 1840, Hall opines on the financial impact of what she terms “a whiskey-drinking habit”: “It spent TIME, and it spent what well-managed time always gives, MONEY.”^68^ The protagonist of the tale attempts to convince his friend of the wisdom of this maxim by confessing the lurid details of his shameful past:

“… eighteen months ago, as you well know, I was in rags—never at my work of a Monday, and seldom on a Tuesday. My poor wife, my gentle patient Mary, often bore hard words, and, though she will not own it, I fear still harder blows when I had driven away my senses. My children were pale, half-starved, naked creatures disputing a potato with the pig.”^69^

In his own personal recollections, the drunkard reinforces the shame of his addiction by reducing his children to naked “creatures” who occupy the same position as swine. Public testimonies from ex-drinkers about their apparently debauched previous lives were a tactic of moral suasion commonly utilized within the temperance movement,^70^ and pamphlets and periodicals often contained a mixture of real and fictionalized “confession” narratives that attempted to warn readers about the dangers of drink.^71^ Hall emphasizes the importance of divulging the details of one’s drinking habits to a moral authority throughout her work—in her short story “Good Spirits and Bad,” for example, a drunkard is portrayed unfavorably for his refusal to “confess” his transgressions to his sober wife.^72^ However, in “The Real Temperance Cordial,” the reformed drunkard’s confession is not quite enough to convince his friend of the benefits of abstention, and so he proudly exhibits his home, which is now the very picture of domestic harmony and industry: “The house was clean, the children rosy, well dressed, and all doing something.”^73^ The protagonist’s convenient omission of any of the difficulties he may have faced in renouncing alcohol helps to convey the beguiling idea that sobriety—and, by extension, financial security—can be attained merely through a strong exercise of the will. In its promotion of an unrealistically idealized version of lower-class familial bliss, “The Real Temperance Cordial” exemplifies the weaponization of domestic happiness that was, as Begiato points out, typical of didactic fiction in this era,^74^ and purports to provide a solution for the poverty that was then rampant in Ireland.

The emphasis placed on family life in both its abject and idealized state points to the idea that the drinker’s shame was reflected and magnified by the physical condition of his wife and children. Medical writers often made use of this idea by referring to the drinker in terms of his communal identity—Wilson’s *Pathology of Drunkenness* (1855), for example, asserted that no drunkard could avoid “spread[ing] somewhere over others the burden of his poverty, the poison of his example, the dispeace of his brawls, the shame of his degradation, and the evil and suffering of his violence.”^75^ A version of “Good Spirits and Bad,” which appeared in the 1844 edition of Hall’s *Sketches of Irish Character*, aligns with the previous example in its unflinching portrayal of the shame of the lower-class male drinker who cannot provide for his family.^76^ Despite frequent attempts to drown his cares with drink, Roney Maher remains keenly aware of his descent from being a “respected” worker to a “degraded” individual^77^ and laments the share his family must take in his degradation. Roney’s children are forced to beg in the streets “for a morsel of food” while his wife can “hardly keep the rags together that shrouded her bent form”—a deliberately provocative allusion that appears to equate poverty with immodesty or indecency.^78^ Another version of this story, published over three decades later in Hall’s collection *Boons and Blessings: Stories and Sketches to Illustrate the Advantages of Temperance* (1875) under the title “It’s Never Too Late,” depicts Roney Maher “without money or credit” as a result of his drinking, and his children are consequently forced to content themselves with a “scanty feast” of potatoes for their evening meal.^79^ This image would have had a particular resonance after the Famine, making the association of starvation with disgrace especially bleak in this context. The different editions of the story therefore demonstrate the consistency of Hall’s didactic arguments—while she may have revised her work in later decades, she did not falter in holding up the drunk, impoverished lower-class man as a shameful example of inadequate manhood. Her stories demonstrate the not-so-subtle transmission of the message that the poor bore responsibility for their poverty and could overcome it only if they aligned themselves with middle-class notions of manliness, industry, and temperance.

## The Childish Brute

The financial hardship and destitution that, according to temperance reformers, was inevitably endured by the family of the lower-class male drinker paled in comparison to their proximity to physical violence. Temperance writers dwelt on the vicious outbursts of temper that supposedly characterized the drinker’s emotional displays while also presenting episodes of weepy sentimentality that further emphasized the unmanly nature of excess. William Carleton, who can be categorized alongside Hall as a chronicler of the Irish peasantry, although his reputation endured long after Hall’s began to wane, utilizes scenes of tears and terror in this fashion in his temperance novel *Art Maguire, or The Broken Pledge* (1845). Originally written as a tract for Charles Gavin Duffy’s Library of Ireland, the novel was intended for an Irish readership^80^ and concludes with a plea addressed to teetotalers and drinkers alike that they avoid following the eponymous protagonist’s path in life. Born in a small town in county Tyrone in 1794, Carleton was the son of a Catholic smallholder and, despite his conversion to Protestantism in his twenties, was for this reason regarded as a valid authority on the lives of the peasantry.^81^ Carleton’s work expresses a deep interest in the habits and rituals of the lower classes, but, as Barry Sloane argues, he consistently endorses the idea that Irish people need to cast off their idiosyncrasies (like their reputed propensity to indulge in raucous drinking sprees) in order to gain respect and legitimacy among the English.^82^

As I shall go on to demonstrate, *The Broken Pledge* is highly medicalized in its emphasis on embodiment and its references to contemporary theories of alcohol use that affirm the stereotype of the inherently drunken, lower-class Irish male. On the surface, the novel follows a standard temperance plot: Carpenter Art Maguire terrorizes and impoverishes his family in the process of becoming a drunkard and, despite making several attempts at reform, eventually dies a lingering death in a workhouse. It is somewhat difficult to ascertain Carleton’s overarching message, diverging as he does from the simplistic idea endorsed by Hall and other authors that temperance merely requires a strong desire to reform. The narrative warns that even “a fine, generousminded, and affectionate young man”^83^ like Art can become ensnared by alcohol, brutalized beyond recognition, and, ultimately, rendered powerless to resist the temptation to drink, thus perpetuating a notably hopeless message about the nature of addiction. In taking this pessimistic stance, Carleton’s novel shares with medical treatises the idea that drinkers could be compelled by a “well-nigh irresistible impulse” that overpowered the desires of even “the kindest souls, the most unselfish spirits.”^84^ In fact, a warm or sensitive disposition was actually believed to hinder the drinker in his path to sobriety. Follen Palmer argued that while “inebriates” were “weak,” they were rarely wicked because: “If they were wicked they would not remain drunkards, for uniform wickedness implies a certain amount of will force, which is all that the inebriates ordinarily require for a cure.”^85^ In *The Broken Pledge*, Art’s lack of willpower is similarly linked to his very amiability, which is portrayed as an inherently Irish trait that, when ignited by drink, places the would-be sober man at a disadvantage. After several pages of detailed exposition describing Father Mathew’s crusade and its avid response among the lower classes, Carleton writes:

When we also consider the natural temperament of the Irishman, his social disposition—his wit, his humour, and his affection—all of which are lit up by liquor […] when we think upon the poverty, the distress, and the misery which too generally constitute his wretched lot, and which it will enable him, for a moment, to forget […] we can see how many temptations our countrymen had to overcome in renouncing it as they did.^86^

While demonstrating recognition of the possibility that the lower classes drank to escape the hardships of poverty rather than incurring poverty on themselves through their heedless consumption, Carleton nevertheless promotes a view of the Irish peasantry as being almost biologically predisposed to drunkenness. Moreover, he situates the lower-class man within a culture that not only uses alcohol as a panacea in times of hardship but that also views drinking as an integral part of celebratory rituals. Carleton explains the effect of this “festive spirit” on Art’s attempts at sobriety: “Whenever his friends were met together, whether at fair, or market, wedding, christening, or during the usual festivals, it is certain that a glass of punch or whiskey never crossed his nose that he did not feel a secret hankering after it.”^87^ Thus the reformed Irish drunkard is placed in a predicament that is almost impossible to navigate, as celebration and mourning both lead to drink, and nature and nurture conspire against him.

Throughout the novel, Carleton takes a firm stance against the normalization of drinking culture by portraying drunkenness as a shameful state that transforms the drinker into a “poor unmanly sot” who loses control over his faculties.^88^ One morning, Art arrives late to work “considerably wrecked and debilitated” after drinking heavily the night before and vainly struggles to compensate for lost time with “tremulous and unsteady hands.”^89^ He is therefore physically unable to take part in the industrious activity expected of a man of his station—a problem that worsens with each escalation of his drinking, until he feels as though he is “but a child in the strong grasp of his own propensities.”^90^ Art’s embarrassing association with childishness is reinforced by his drunken emotional display on the night of his daughter’s christening, when he descends into a state of tearful sentimentality as he rhapsodizes over the love he bears for his wife. Although Art protests that he is “not a bit ashamed of these tears,”^91^ his reiterations of this statement signify an obvious attempt to convince *himself* of his lack of shame, and he appears as a somewhat ludicrous, and certainly emasculated, figure throughout the episode. Evidence of the continued use of this literary device in later decades can be seen in Bram Stoker’s “The Primrose Path” (1875), which depicts a hungover Jerry O’ Sullivan who tearfully allows his wife to rock his head on her breast “as a while ago she had rocked her baby’s.”^92^ The scene is disconcerting, as Jerry’s rapid fall from the masculine role of protector and provider to the helplessness (and, eventually, the economic uselessness) of a small child is portrayed as embarrassing and distasteful. Doctors commented on the male drinker’s tendency to exhibit what were regarded as inappropriate signs of emotion, too. T. L. Wright, for example, rather disdainfully stated that “Any tale of sympathy, or even a train of sympathetic ideas, will cause the tears of maudlin emotion to stream from the eyes.”^93^ Thus, even the apparently innocuous signs of intoxication were held up for the reader’s scrutiny and resulting scorn.

Art reaches new depths of abjection as *The Broken Pledge* progresses, becoming totally vicious, abusive, and impervious to the pleading of his starving wife and children, who are forced to beg for scraps of food. In fact, he is barely even a cognizant human, as the reader is informed that Art’s mind is “likely soon to be free from thinking,” for he has sunk “to the lowest and most pitiable state of degradation.”^94^ Art is therefore not only emasculated but also dehumanized by his addiction, rendered an unthinking, unfeeling brute motivated only by his thirst for alcohol. However, Art’s violence toward his family is portrayed not as deliberate abuse but rather as an almost incidental, and certainly uncontrollable, consequence of his drinking. By emphasizing the danger that intoxicated fathers and husbands posed to those closest to them, temperance writers played on familial attachments as they attempted to impress their readers with the sense that a drunk body was shamefully uncontrolled, capable of committing brutal acts it would wholeheartedly condemn while sober. In their analysis of domestic violence in mid-nineteenth-century literature, Kate Lawson and Lynn Shakinovsky argue that the Victorian medical model of physical abuse asserted that “alcoholism causes domestic violence through a mysterious process” that renders the drinker wholly unable to control his furious and irrational outbursts.^95^ Alcohol’s “mysterious” facilitation of violence is made evident in the contrast Carleton draws between the happy and companionable nature of Art’s marriage during his periods of sobriety with the “violent and brutal” scenes that take place once he is fully immersed in drunkenness.^96^

Incidents of physical abuse are rarely described explicitly,^97^ but the reader is informed that Margaret regularly bears “the foul mark of her husband’s brutality” on her face.^98^ As Mary O’Dowd and Maria Luddy outline, a wife’s bruised or cut body could provide a powerful testimony within the Irish court system,^99^ but Margaret is far from having any desire to prosecute Art. In fact, she forbids her children to speak of his violence, stating, “‘your poor father never beat me, only when he didn’t know what he was doin.’”^100^ The “doublethink” Margart participates in here (Art *never* beat Margaret, *except* when he was drunk) encapsulates two pervasive ideas of temperance reform: It was the drink and not the drunkard who bore responsibility for violence, and it was the wife’s duty to be endlessly passive in the face of said violence.^101^ The implication that the wife of a drinker should not expose evidence of abuse emerges again in “The Primrose Path,” when Katey appears at her husband’s court hearing with the euphemistically termed “signs of ill-usage” on her face, and, upon noticing her bruises, the magistrate imposes a higher sentence on Jerry in an effort to “put down wife-beating.”^102^ While Katey is not explicitly framed as being at fault here, there is a strong implication that her exposure of domestic violence only perpetuates the suffering of her family, who are plunged into deeper poverty as a result of Jerry’s prolonged absence from work. While the violent drinker is portrayed as unmanly, then, he is condemned not necessarily for the abuse itself but for making the choice to drink, which disrupts the traditional dynamic between a protective provider and his submissive wife.

Eventually, Art becomes so brutalized that he delivers a blow to his favorite son Atty, which “kill[s] his mind” and renders him “a mild, placid, harmless idiot.”^103^ While Atty’s intellect is blunted as a direct result of Art’s violence rather than being due to any inherited predisposition, it is interesting to read this incident in light of theories of heredity and degeneration that would take a strong foothold by the end of the nineteenth century. The high demand on asylums and workhouses in Ireland, as well as the high proportion of Irish inmates in British institutions, would then be used as evidence to argue that bodily and mental failing widely afflicted the Irish population, and these issues were presumed to be exacerbated by drink.^104^ Writing on the “brain mischief” that he argued was likely to be suffered by the children of drunkards, prominent alienist John Charles Bucknill mentions heredity before going onto reference neglect and “the influences of a turbulent home,”^105^ grouping together two disparate causes of mental injury under one umbrella. It is unclear whether Bucknill refers here to violent incidents of the kind that renders Atty an idiot in *The Broken Pledge*, or whether he speaks of the cumulative traumatic effect that exposure to violence has on young minds. However, he appears to align with Carleton in his expression of the belief that, one way or another, drunkenness results in child idiocy through exposure to the “turbulence” perpetuated by parents who drink.

Doctors complained that their warnings about child endangerment were likely to fall on deaf ears: Speaking to the *Royal Academy of Medicine in Ireland* in 1892, E. Cosgrave MacDowel lamented the fact that even when fathers became convinced that their drinking was “destroying the happiness of their wives and children,” they were seemingly unable to alter their habits.^106^ Art is similarly cognizant of the harm he is inflicting on his family, at one point exclaiming: “‘What have I brought you to, my darlin’ wife and children, that I loved a thousand times betther than my own heart?’”^107^ However, he remains unable to achieve permanent sobriety, and his brief stints of abstinence do little to restore “the healthy and powerful constitution which he had lost.”^108^ Despite the sympathy for Art’s abjection that Carleton attempts to invoke in the reader here, he is quite relentless in his portrayal of the drunken lower-class man as a savage or demon who bears no resemblance to the manly head of the household upheld by society as the ideal protector and provider. By painting all forms of bodily “excess,” including drunkenness, tears, and violence, as indicators of unmanliness, temperance writers confined the acceptable display of masculinity to a narrower sphere, reinforcing the idea that any kind of rebellion (political or social) was to be avoided. Carleton applies this message to the Irish context by setting out the cultural, political, and biological factors that supposedly combine to make Irishmen peculiarly prone to drunkenness, encouraging the lower classes to conform to traditionally gendered, strictly regulated standards of work and celebration.

## Unnatural Mothers

Nineteenth-century medical works on alcohol consumption tended to argue that drunkenness was a social issue more commonly endured, rather than perpetuated, by women. William Marcet, for example, stated that he had only ever seen “four or five cases of chronic alcoholism” in total among the entire cohort of his female patients.^109^ If women supposedly did not struggle to regulate their alcohol consumption in the same way that men did, then it was seen as their duty to protect their homes (and their men) from the perils of drink. Early nineteenth-century British conduct literature accordingly reiterated the idea that “a well-chosen word from wife to husband might discourage overindulgence,” as Pam Lock points out,^110^ and Irish temperance literature also placed a heavy-handed emphasis on this moral obligation. Consider, for example, Hall’s desire that “all Irish maidens” would endeavor to prove to their menfolk that “‘the least taste in life’ is a great taste too much!— that ‘only a drop’ is a temptation fatal if unresisted.”^111^ Although Elizabeth Malcolm has argued that women did not play an active independent role in the Irish temperance movement,^112^ Luddy demonstrates that women’s philanthropy had a significant impact on society, not only because these groups provided material aid to the poor but also because their campaigns helped to shape the values of Irish people.^113^ Like other philanthropists of the period, temperance reformers sought to change personal habits and belief systems, rather than socioeconomic structures, through their gentle persuasion and example.^114^ Mothers, in particular, were exhorted to lend their efforts to the temperance movement because they were supposedly accustomed to inculcating their children with the value of self-restraint, and the characterization of female temperance reformers as motherly figures had the added benefit of mitigating their radicalism.^115^

The idealization of a kind of philanthropic motherhood, wherein a woman takes responsibility for the less fortunate as well as for her own family, can be viewed as part of a wider social debate surrounding mothers and drinking. Holly Dunbar points out that the mainstream Irish nationalist press drew a paradoxical association between women as virtuous examples to their families and as worse drinkers than men if they consumed alcohol—a double standard that was linked to concerns over degeneration and the maintenance of public order.^116^ Similarly, in Britain, women’s drinking was not interpreted or treated as a “disease” but was rather grouped with other manifestations of femininity that were considered highly disruptive and disreputable, like prostitution.^117^ Working-class women became the focus of concerns about the respectability of public spaces, and those who drank were criticized for supposedly compromising their ability to produce healthy off-spring as well as violating their duty to serve as a moral example. Dorota Dias-Lewandowska and Lock argue that the female drinker therefore represented “a multiple threat to family and community,”^118^ and this idea is clearly present in Irish temperance literature. As will become clear in this section, Irish temperance writers attempted to convince their readers of the idea that, just as the male drinker was shamefully emasculated by drink, so too was the female drinker rendered embarrassingly unfeminine or grotesque, posing a threat to her loved ones and to society at large. In doing so, they drew on international discussions about women’s drinking and applied them to Ireland, perhaps due to their awareness that the country’s reputation was particularly implicated in concerns about lower-class degeneration and disorder.

We can see evidence of this rhetoric in the work of Marion Clarke (1841–1928), the daughter of a Presbyterian landowner from County Down who produced 14 moralistic novels over the course of her career and occasionally published poems in temperance periodicals under the alias “Miriam Drake.” While the temperance movement generally depicted women as the victims (or the reformers) of drunk men rather than as drinkers themselves,^119^ Clarke provides us with the unusual example of an upper-class female character who, over the course of the novel, exists as a victim, a drinker, and a reformer.^120^ *Oughts and Crosses: A Novel With A Moral* (1872) depicts the interrelations of aristocratic Protestant families in the fictional village of Loughamore, detailing the efforts of certain pious and abstinent characters to eradicate drunkenness from the region.^121^ Its focus on the danger of prescribing women alcoholic beverages as “stimulants” reflects a then ongoing debate over the medicinal value of wine and spirits, which, as Thora Hands demonstrates, was heavily influenced by ideas pertaining to the moral implications of drinking.^122^ Moreover, the novel can be read as part of a broader moral panic surrounding the possibility that women might be secretly consuming alcohol purchased from spirit grocers, which David Beckingham argues propagated a kind of moral scrutiny among middle-class British women.^123^ While doctors generally regarded alcohol as medicinal in moderation but as morally reprehensible in excess,^124^ Clarke sets out to demonstrate not only that there is no medicinal benefit to alcohol but also that “moderation” is an impossibly slippery concept that is highly responsive to the drinker’s self-delusion about how much she drinks.

Accordingly, the novel strongly rejects medical authority when it comes into conflict with temperance. In an explicit assertion of her own moral values over medical advice, the teetotal heroine Miriam confronts the doctor when he prescribes alcohol as a preventative medicine, stating: “I cannot see how stimulation, which is itself feverishness, can prevent fever.”^125^ However, *Oughts and Crosses* contains no radical endorsement of female agency, as it emphatically insists on the virtue of female submission in marriage and frames any subversion of gender norms as shameful and disturbing (at one point a male teetotaler piously exclaims that “an effeminate man is simply disgusting”).^126^ The novel’s main plot traces the development of the unhealthy marital dynamic between wealthy young Philip Seguin and his naive wife Mattie, explicitly framing alcohol as a danger to marital satisfaction because it defeminizes the wife and emasculates the husband. The reader is informed that Mattie’s “extreme femininity” activates Philip’s sense of “manliness,” and he is consequently gentle to her when sober, but “whenever his passions were inflamed by drink they got the better of his reason, and he would grow savage.”^127^ In other words, Mattie’s ostensibly natural, appealing femininity does not have the proper effect when Philip is rendered unmanly by drunkenness, and his verbal abuse eventually escalates to physical violence. However, Mattie’s victimhood is complicated by her own dependence on alcohol, which, although it stems from her innocent adherence to the doctor’s advice, leads her to neglect her duty as wife and mother in rather spectacular ways. Doctors frequently infantilized women in their approach to alcohol consumption,^128^ and we see a literary illustration of this medical tendency when, against her better judgment, Mattie “bow[s] dutifully” to her doctor’s injunction that she consume “a glass or two of light wine in the day” to keep up her strength.^129^ What is portrayed as a natural feminine distaste toward drinking is gradually eroded, for “taking alcohol herself so constantly—as medicine, of course—had somewhat blunted the dread of seeing Philip take it.”^130^ Eventually, Mattie is unable to limit herself to her doctor’s prescription and begins to consume port and “quantities of brandy,”^131^ beverages that were regarded as being far more dangerous than beer or wine.^132^ Consequently, she becomes “sharp and querulous” with her husband, until she is unable to refrain from “answering every hasty word of Philip’s with a bitter one, which too often led to others from him, till hysterics and brandy ended the controversy.”^133^ Instead of submitting meekly to her husband, Mattie rebels against Philip’s cruel treatment of her, and the situation continues to escalate until he takes refuge in the brandy bottle—and perhaps she does likewise. Mattie therefore loses a valuable opportunity to reform her husband through softness and passivity, but there is an inconsistency here, because it is Mattie’s passive obedience to her doctor’s orders that has landed her in this predicament. The narrative therefore appears to place more blame on the doctor than on the upper-class female drinker who is seemingly predisposed to submit to male authority, but the specter of indecent or degraded womanhood nevertheless looms large throughout the text.

Even when her spirits are elevated by alcohol, Mattie is unable to exhibit the proper degree of femininity required from an upper-class wife. In one scene, Mattie welcomes her visitors in “a tumult of glad excitement” after privately taking her “forenoon allowance of wine,” and she begins to talk “incessantly […] with great glee […] descanting on the baby even more volubly than usual.”^134^ Thus, Clarke makes the point that even a moderately fuddled mother is irresponsible with her child. Even more shocking to her visitors, however, is Mattie’s inappropriate conversation, as she jokes that the local distiller is courting her aunt and babbles “more vivaciously than any French woman.”^135^ Her childhood friend Moses is “rather disgusted” at her behavior and tells Mattie not to laugh so much after taking wine, for “if people did not know they would think you were tipsy.”^136^ While Mattie’s drinking is just barely rendered respectable by the doctor’s pronouncement that it will benefit her health, her laughter and vivacity threaten to topple this shaky justification through their indication that drinking is in fact a source of pleasure for her. While Mattie is neither explicitly coarse nor violent in this scene, she is embarrassingly indelicate, and the episode illustrates that even moderate drinking, performed at home and with a doctor’s approbation, can lead to shameful and, crucially, physically visible signs of intoxication.

The incompatibility between drinking and responsible motherhood is further highlighted in a gruesome episode wherein a drunken nurse smothers Mattie’s first baby in her sleep.^137^ Not only does this tragedy provide the reader with a stark demonstration of the dangers of female drinking, but it also foreshadows Mattie’s own act of child murder. When her second son Charley falls ill with a severe attack of croup, Mattie gulps down two glasses of wine in a panic before attempting to administer his medicine. Clearly muddled by the alcohol, Mattie accidentally gives her son a large dose of strychnine and he dies in excruciating agony—a trope that was often employed by temperance writers to emphasize the idea that alcohol had no place in medical practice.^138^ The shame she feels after being confronted with clear evidence of her unfitness for motherhood causes her to enter a state of “black despair,”^139^ and although the doctor mercifully keeps the secret of Charley’s real cause of death to save Mattie from prosecution, she experiences a bout of insanity that twists her motherhood into something that is at once childish and grotesque. Incoherent, distressed, and evidently hallucinating—symptoms that align her with contemporary depictions of *delirium tremens*—Mattie clings to a doll and “crooned to it all night, but sometimes she would break off suddenly screaming” for alcohol.^140^ However, she is redeemed through Miriam’s watchful nursing, and upon her recovery, she becomes a temperance reformer who persuades poor female drunkards to take the pledge, remaining “peculiarly tender [with them …] long after other lady visitors had given them up in disgust.”^141^ Clarke therefore portrays Mattie’s philanthropic endeavors essentially as a form of nonbiological mothering that marks an attempt to atone for the death of her son. Following his wife’s example, Philip embraces temperance, and the pair goes on to have more children, who “never having tasted wine or strong drink, are saved from the fatal craving that their father imbibed with his mother’s milk.”^142^ Mattie’s reformation therefore grants her the power to subvert alcoholic heredity, and the novel exists as an intriguing example of a text that, while highly medicalized, nevertheless undermines the doctor’s authority by elevating the example of the teetotal woman —but in such a way that her adherence with traditional standards of femininity cannot be called into question.

Upper-class female drunkards like Mattie are highly unusual within Irish temperance literature. Far more common is the figure of the lower-class woman exemplified in Hall’s “Bridget Larkins—The Mountain Lass.” The story places a similar emphasis on the female drinker’s inevitable violation of the sacred duty of motherhood while also dwelling on the social chaos that the drunken Irish woman could supposedly cause. When fishwife Bridget drinks whiskey, she transforms into a wild creature who is “half maniac, half demon” and displays an unfortunate propensity to “hurl fish after fish” at her customers.^143^ However, the reader is informed that Bridget is “a kind and generous person” when sober, and on one occasion, “while more than half tipsy,” she swims into the quay to rescue the child of a rival fishwife who is “more intoxicated than herself”—an overtly masculine yet brave and selfless act that demonstrates her humanity is still present even in the midst of her drunken defeminization.^144^ Hall’s attitude toward this character appears to be one of amused tolerance and even sympathy, but she nevertheless hammers home the point that Bridget’s better qualities are overshadowed by her drinking. While Bridget initially takes pride and comfort in the fact that she has never “riz [her] hand to a child” in one of her drunken rages, this consolation is eventually denied her after she throws a stone at a taunting boy and almost kills him.^145^ She is then reduced to a pitiful and degraded state of “grovell[ing]…in [her] miserable hut,”^146^ rendered totally abject after committing such an unmaternal act. All ends well for Bridget, however, as the boy survives, and she ends by taking and keeping Father Mathew’s temperance pledge—in stark contrast to Art Maguire’s failure. The story tritely concludes with the moral that it is always possible for individuals to be “reclaimed at the eleventh hour [… and become] blessings to all within their sphere,”^95^ aligning the narrative with the standard temperance trope that portrays the shame of committing violence against a child as a catalyst for the drunkard’s reform.

Hands argues that concern over female drinking in Victorian Britain focused on public drunkenness among lower-class women and on private drinking among upper-class women,^147^ and these stories provide literary evidence that this distinction was also made in 1870s Ireland. While Mattie privately imbibes brandy and wine and convinces herself of their medicinal properties, Bridget publicly knocks back large quantities of whiskey and apparently requires no justification for doing so. Her lower social status grants her a certain level of freedom, but it also renders her more masculine than the timorous, ladylike Mattie, whose behavior never escalates into physical violence. However, Mattie and Bridget both serve to illustrate the misery and shame of female intemperance, and both are reformed only after rendering significant harm to a child, cementing the contemporary medical connection drawn between alcohol consumption and degenerated maternity. These characters also reflect ideas pertaining to the supposedly disruptive potential of the female drinker: Mattie’s neglect of her philanthropic duties and Bridget’s chaotic outbursts in the fish market both highlight the idea that sobriety and submission were crucial performances for women who wished to promote the welfare of Ireland, regardless of their class. However, as with Hall’s insistence that poverty was attributable to personal failure and Carleton’s argument that the Irish temperament was particularly “lit up by liquor,”^148^ the depiction of the idealized Irish woman as enshrined within the domestic space (save for her philanthropic excursions) is rather unconvincing. As Lloyd argues, Irish attempts to replicate the Victorian “Angel in the House” ideal were “at best utopianist” because women were an integral part of the industrialized workforce in Belfast and Dublin and performed extensive farm labor in rural areas.^149^

The significant body of temperance literature produced by Irish writers throughout the nineteenth century serves to illustrate the gendered nature of the moral and medical scrutiny to which the drinker was subjected. In accordance with international stereotypes, Irish temperance writers depicted the figure of the drunkard as necessarily impoverished and unproductive, as violent or abusive, and as a neglectful parent, in ways that aligned with prevailing medical theorizations of the issue. These manifestations of the drunkard as a literary trope indicate that intoxication was clearly associated with gender subversion and contributed to a perception of the supposed unmanliness of the lower-class Irish male, which, I have shown, was propagated by various iterations of the temperance movement among Protestants and Catholics. The persistence of these stereotypes over the course of the period surveyed indicates that “drunkenness,” and its associated disruptions to public order, continued to be a source of concern to reformers and commentators throughout the nineteenth century. Of course, temperance authors may also have been aware that these debauched caricatures held a voyeuristic appeal for readers who enjoyed being repeatedly titillated by tales of drunken abjection and degraded Irishness. In appearing to endorse the idea that Irish people had an unfortunate propensity toward drunkenness (whether because of their disposition, misfortune, or supposedly weak intellect) that could only be overcome through a strenuous adherence to British standards of respectability, these writers likely reinforced ideas of Irish people as being perpetually drunken and served to make class divisions more deeply entrenched.

## Footnotes

1 “Manliness,” *The Irish Temperance Star* 1, no. 4 (1866): 62–63.

2 ”Manliness,” 62.

3 ”Manliness,” 63.

4 Charles Wilson, *The Pathology of Drunkenness: A View of the Operation of Ardent Spirits in the Production of Disease* (Edinburgh, 1855), 60.

5 Charles Follen Palmer, *Inebriety: Its Source, Prevention, and Cure* (Union Press, 1897), 39.

6 Henry Osborne, “Prevention and Elimination of Disease, Insanity, Drunkenness and Crime—A Suggestion.” *Journal of the Statistical and Social Inquiry Society of Ireland* 10 (1894): 85.

7 Joanne Begiato, *Manliness in Britain: 1760*–*1900* (Manchester University Press, 2020), 68–100; Gale M. Temple, “‘Affrighted at the Eager Enjoyment’: Hawthorne, Nymphomania, and Medical Manhood.” *Textual Practice* 28, no. 3 (2014): 405–25.

8 John Edward Walsh, *Sketches of Ireland Sixty Years Ago* (William S. Orr & Co., 1849), 60.

9 Lucy Cogan, “Drunkenness, Compulsion, and the Disintegration of the Self: Erasmus Darwin’s Theory of Ebrietas in the Writings of Maria Edgeworth,” in *Life, Death, and Consciousness in the Long Nineteenth Century*, ed. Lucy Cogan and Michelle O’Connell (Springer International Publishing, 2022), 31–53, at 39.

10 Lucy Cogan, “Dorothea Du Bois’s *Theodora*: Drinking, Dueling, and Domestic Violence in Eighteenth-Century Irish Literature,” *The Social History of Alcohol and Drugs* 37, no. 2 (2023): 210–38, at 217.

11 James Kelly, “The Consumption and Sociable Use of Alcohol in Eighteenth-Century Ireland,” *Proceedings of the Royal Irish Academy, Section C: Archaeology, Celtic Studies, History, Linguistics, Literature* 115 (2015): 219–55, at 243.

12 John Tosh, “Gentlemanly Politeness and Manly Simplicity in Victorian England,” *Transactions of the Royal Historical Society* 12 (2002): 455–72, at 458.

13 Norman Vance, *The Sinews of the Spirit* (Cambridge University Press, 1985), 25.

14 Begiato, *Manliness*, 94.

15 Gwen Hyman, “‘An Infernal Fire in My Veins’: Gentlemanly Drinking in the Tenant of Wildfell Hall,” *Victorian Literature and Culture* 36, no. 2 (2008): 451–69, at 457.

16 L. Perry Curtis, “The Four Erins: Feminine Images of Ireland, 1780–1900,” *Eire-Ireland* 33, no. 3 (1998), 72, 89.

17 Mary J. Corbett, *Allegories of Union in Irish and English Writing, 1790*–*1870: Politics, History, and the Family from Edgeworth to Arnold* (Cambridge University Press, 2000), 3.

18 Joseph Valente, “The Myth of Sovereignty: Gender in the Literature of Irish Nationalism,” *Elh* 61, no. 1 (1994): 189–210.

19 Corbett, *Allegories*, 7.

20 Valente, “Myth,” 192.

21 Valente, “Myth,” 192.

22 ”Manliness,” 62–63.

23 David Lloyd, “Counterparts: Dubliners, Masculinity, and Temperance Nationalism,” in *Burning Down the House*, ed. Rosemary Mangoly George (Routledge, 1998), 150–69, at 154.

24 Jessica Warner, *Craze: Gin and Debauchery in an Age of Reason* (Random House, 2003).

25 Roy Porter, “The Drinking Man’s Disease: The ‘Pre-History’ of Alcoholism in Georgian Britain,” *British Journal of Addiction* 80, no. 4 (1985): 385–96, at 388, 391.

26 James Nicholls, *The Politics of Alcohol: A History of the Drink Question in England* (Manchester University Press, 2011), 123.

27 Virginia Berridge, “Morality and Medical Science: Concepts of Narcotic Addiction in Britain, 1820–1926,” *Annals of Science* 36, no. 1 (1979): 67–85, at 76.

28 Berridge, “Morality,” 77.

29 Wilson, *Pathology*, 216.

30 Norman Kerr, *Inebriety: Its Etiology, Pathology, Treatment and Jurisprudence* (J. S. Tait & Sons, 1888), 3.

31 Norman Kerr, “The Relation of Alcoholism to Inebriety,” *British Medical Journal* 1, no. 1434 (1888): 1320–22, at 1320.

32 Alice Mauger, “‘The Holy War Against Alcohol’: Alcoholism, Medicine and Psychiatry in Ireland, c. 1890–1921.” *Healthy Minds in the Twentieth Century: In and Beyond the Asylum*, ed. S. J. Taylor and A. Brumby (2020), 17–52, at 21.

33 Mauger, “Holy War,” 20.

34 William Saunders Hallaran, *Practical Observations on the Causes and Cure of Insanity* (Edwards and Savage, 1818), 27.

35 Cogan, “Dorothea,” 238.

36 Colm Kerrigan, “The Social Impact of the Irish Temperance Movement, 1839–1845,” *Irish Economic and Social History* 14, no. 1 (1987): 20–38, at 20.

37 Mauger, “Holy War,” 37–38. Mauger’s chapter refers mainly to data from the 1890s, and so the pledge would not have related to Father Matthew in this instance. However, it nevertheless serves as a reminder that pledge-takers did not necessarily remain abstinent from alcohol.

38 Kerrigan, “Social Impact,” 21.

39 Elizabeth Malcolm, “The Catholic Church and the Irish Temperance Movement (1838–1901),” *Irish Historical Studies* 23, no. 89 (1982): 1–16, at 14.

40 Malcolm, “Catholic Church,” 15.

41 Elizabeth Neswald, “‘The Benefits of a Mechanics’ Institute and the Blessing of Temperance’: Science and Temperance in 1840s Ireland,” *The Social History of Alcohol and Drugs* 22, no. 2 (2008): 209–27, at 219.

42 Nicholls, *Politics*, 104, 107.

43 Lloyd, “Counterparts,” 99.

44 Joseph Nugent, “The Sword and the Prayerbook: Ideals of Authentic Irish Manliness,” *Victorian Studies* 50, no. 4 (2008): 587–613, at 608.

45 Diarmaid Ferriter, *A Nation of Extremes: The Pioneers in Twentieth Century Ireland* (Irish Academic Press, 1998).

46 Brad Beaven and John Griffiths,”Creating the Exemplary Citizen: The Changing Notion of Citizenship in Britain 1870–1939,” *Contemporary British History* 22, no. 2 (2008): 203–25, at 212–13.

47 James H. Murphy, *Irish Novelists andthe Victorian Age* (Oxford University Press, 2011), 5.

48 See Sally Shuttleworth, *The Mind of the Child: Child Development in Literature, Science, and Medicine, 1840*–*1900* (Oxford University Press, 2010); and Cogan, “Drunkenness.”

49 Claire Connolly, *A Cultural History of the Irish Novel, 1790*–*1829* (Cambridge University Press, 2011), 122.

50 Helen M. O’Connell, *Ireland and the Fiction of Improvement* (Oxford University Press 2016), 13.

51 Niall Ó Ciosáin, *Print and Popular Culture in Ireland: 1750*–*1850* (Lilliput Press, 2010), 154.

52 Elizabeth Tilley, “The *Dublin Penny Journal* and Alternative Histories,” in *Literacy, Language and Reading in Nineteenth-Century Ireland*, ed. Rebecca Anne Barr, Sarah Anne Buckley, and Muireann Ó Cinnéide (Liverpool University Press, 2019), 87–103, at 87–88.

53 Tilley, “*Dublin Penny Journal*,” 100.

54 Ó Ciosáin, *Print*, 172.

55 O’Connell, *Improvement*, 13, 103, 167.

56 Karen Sanchez-Eppler, “Temperance in the Bed of a Child: Incest and Social Order in Nineteenth-Century America,” *American Quarterly* 47, no. 1 (1995): 1–33, at 15.

57 Steven Earnshaw, *The Existential Drinker* (Manchester University Press, 2018), 45. Hence-forth I will use the pejorative term “drunkard,” which was widely used in nineteenth-century medical and temperance works, when discussing the drinker’s characterization as a literary figure.

58 Annemarie McAllister, “*Onward*: How a Regional Temperance Magazine for Children Survived and Flourished in the Victorian Marketplace,” *Victorian Periodical Review* 48, no. 1 (2015): 42–66, at 43.

59 Christine Kinealy, *This Great Calamity: The Irish Famine, 1845*–*52*. (Gill & Macmillan, 2006), 196, 39.

60 Catherine Cox, *Negotiating Insanity in the Southeast of Ireland, 1820*–*1900* (Manchester University Press, 2012), 36, 171.

61 James Haughton, “To the Farmers of Ireland,” *Irish Temperance League Journal* 3, no. 9 (1865), 129.

62 David Ross, “Suggested Practical Checks on Excessive Drinking and Habitual Drunkenness,” *Journal of the Statistical and Social Inquiry Society of Ireland* 6 (1870): 397–407, at 400.

63 Kerrigan, “Social Impact,” 21.

64 Anna Pilz, “The Rise of the Woman Writer,” in *Irish Literature in Transition: 1830*–*1880*, ed. Matthew Campbell (Cambridge University Press, 2020), 257–79, at 273.

65 Anna Maria Hall, *Boons and Blessings: Stories and Sketches to Illustrate the Advantages of Temperance* (Virtue, Spalding, and Co., 1875), 24.

66 Hall, *Boons*, 54.

67 Hall, *Boons*, 198.

68 Anna Maria Hall, “The Real Temperance Cordial,” *The Irish Penny Journal* 1, no. 19 (1840): 149–50, at 149.

69 Hall, “Temperance Cordial,” 149.

70 Nicholls, *Politics*, 103.

71 Pam Lock,”George Eliot Writing the Drunken Husband: ‘Janet’s Repentance’ as a Study of Male Violence and Co-Dependence,” *Women*’*s Writing: The Elizabethan to Victorian Period* 28, no. 2 (2021): 250–64, at 251.

72 Anna Maria Hall, *Sketches of Irish Character* (How and Parsons, 1844), 372.

73 Hall, “Temperance Cordial,” 149.

74 Begiato, *Manliness*, 149.

75 Wilson, *Pathology*, 194.

76 *Sketches of Irish Character* was originally published in 1829, but several expanded issues were subsequently released.

77 Hall, *Sketches*, 372.

78 Hall, *Sketches*, 373.

79 Hall, *Boons*, 48, 49.

80 O’Connell, *Improvement*, 44.

81 Barry Sloan, “The Dilemma of William Carleton, Regional Writer and Subject of Empire,” *Romance, Revolution and Reform* 1 (2019): 92–108, at 97.

82 Sloan, “Dilemma,” 101.

83 William Carleton, *Art Maguire; or, the Broken Pledge* (James Duffy, 1845), 45.

84 Kerr, *Inebriety*, 4.

85 Palmer, *Inebriety*, 40.

86 Carleton, *The Broken Pledge*, 157, 158.

87 Carleton, *The Broken Pledge*, 83.

88 Carleton, *The Broken Pledge*, 46.

89 Carleton, *The Broken Pledge*, 114.

90 Carleton, *The Broken Pledge*, 119.

91 Carleton, *The Broken Pledge*, 97.

92 Bram Stoker, “The Primrose Path,” *The Shamrock* 12, no. 434 (1875): 289–365, at 332.

93 T. L. Wright, “Inebriety: A Study upon Alcohol in its Relations to Mind and Conduct,” *Alienist and Neurologist* 3, no. 2 (1882), 10.

94 Carleton, *The Broken Pledge*, 130.

95 Kate Lawson and Lynn Shakinovsky, *The Marked Body: Domestic Violence in Mid Nineteenth-Century Literature* (Suny Press, 2002), 75.

96 Carleton, *The Broken Pledge*, 132.

97 Jerome Nadelbaft identifies a similar authorial reticence regarding descriptions of physical abuse in Antebellum temperance literature. See Jerome Nadelbaft, “Alcohol and Wife Abuse in Antebellum Male Temperance Literature,” *Canadian Review of American Studies* 25, no. 1 (1995): 15–44, at 21.

98 Carleton, *The Broken Pledge*, 135.

99 Mary O’Dowd and Maria Luddy, *Marriage in Ireland, 1660*–*1925* (Cambridge University Press, 2020), 317.

100 Carleton, *The Broken Pledge*, 191.

101 Lisa A. Surridge, *Bleak Houses: Marital Violence in Victorian Fiction* (Ohio University Press, 2005), 30.

102 Stoker, “Primrose Path,” 362.

103 Carleton, *The Broken Pledge*, 241.

104 Catherine Cox, Hilary Marland, and Sarah York, “Emaciated, Exhausted, and Excited: The Bodies and Minds of the Irish in Late Nineteenth-Century Lancashire Asylums,” *Journal of Social History* 46, no. 2 (2012): 500–24, at 513.

105 John Charles Bucknill, *Habitual Drunkenness and Insane Drunkards* (Macmillan, 1878), 34.

106 E. Cosgrave McDowel, “The Control of Inebriates,” *Transactions of the Royal Academy of Medicine in Ireland* 10, no. 1 (1892): 367–79, at 370.

107 Carleton, *The Broken Pledge*, 188.

108 Carleton, *The Broken Pledge*, 198.

109 William Marcet, *On Chronic Alcoholic Intoxication* (Churchill, 1862), 68.

110 Pam Lock,”Death and the Alcoholic: Public Discourses of Alcoholism in Anne Brontë’s *The Tenant of Wildfell Hall*.” *The Social History of Alcohol and Drugs* 29, no. 1 (2015): 29–47, at 30.

111 Hall, *Boons*, 188.

112 Elizabeth Malcolm, ‘*Ireland Sober, Ireland Free*’: *Drink and Temperance in Nineteenth Century Ireland* (Gill and Macmillan, 1986), 176.

113 Maria Luddy, “Women and Philanthropy in Nineteenth-Century Ireland,” in *Women, Philanthropy, and Civil Society*, ed. Kathleen D. McCarthy (Indiana University Press, 2001), 9–28, at 25.

114 Luddy, “Women,” 23.

115 Emma Liggins, “Not an Ordinary ‘Ladies’ Paper:’ Work, Motherhood, and Temperance Rhetoric in the *Woman*’*s Signal*, 1894–1899,” *Victorian Periodicals Review* 47, no. 4 (2014): 613–30, at 617.

116 Holly Dunbar, “Women and Alcohol During the First World War in Ireland,” *Women*’*s History Review* 27, no. 3 (2018): 379–96, at 392.

117 Jennifer Wallis, “A Home or a Gaol? Scandal, Secrecy, and the St James’s Inebriate Home for Women,” *Social History of Medicine* 31, no. 4 (2018): 774–95, at 791.

118 Dorota Dias-Lewandowska and Pam Lock,”Women, Home, and Alcohol: Constructed Façades and Social Norms in Nineteenth-Century Polish and British Representations of Female Drinking Practices,” *Journal of Victorian Culture* 28, no. 2 (2023): 227–42, at 228.

119 Dunbar, “Women,” 393.

120 George Eliot’s “Janet’s Repentance” also depicts the wife of a drunkard as both a victim of abuse and a drinker herself, as Lock demonstrates. See Lock, “George Eliot,” 250.

121 The novel was serialized in *The Irish Good Templar* in 1873.

122 Thora Hands, *Drinking in Victorian and Edwardian Britain: Beyond the Spectre of the Drunkard* (Springer International Publishing, 2018), 96.

123 David Beckingham, “Private Spirits, Public Lives: Sober Citizenship, Shame and Secret Drinking in Victorian Britain,” *Journal of Victorian Culture* 26, no. 3 (2021): 419–34, at 428.

124 Cogan, “Drunkenness,” 50.

125 Clarke, *Oughts*, 158.

126 Clarke, *Oughts*, 322.

127 Clarke, *Oughts*, 244.

128 Dias-Lewandowska and Lock, “Women,” 237.

129 Clarke, *Oughts*, 252.

130 Clarke, *Oughts*, 231.

131 Clarke, *Oughts*, 271.

132 William Marcet’s *On Chronic Intoxication*, for example, promotes “the moderate use of weak alcoholic beverages” like beer and wine but condemns spirits. See Marcet, *Chronic Intoxication*, 9.

133 Clarke, *Oughts*, 252.

134 Clarke, *Oughts*, 271.

135 Clarke, *Oughts*, 271.

136 Clarke, *Oughts*, 271.

137 Clarke, *Oughts*, 238.

138 See, for example, Ellen Wood’s *Danesbury House* (1860).

139 Clarke, *Oughts*, 279–80.

140 Clarke, *Oughts*, 291.

141 Clarke, *Oughts*, 318.

142 Clarke, *Oughts*, 363.

143 Hall, *Boons*, 190.

144 Hall, *Boons*, 190.

145 Hall, *Boons*, 191.

146 Hall, *Boons*, 193.

147 Hands, *Drinking*, 40.

148 Carleton, *The Broken Pledge*, 157.

149 Lloyd, “Masculinity,” 101.

